# A Case Report of Pseudomonas Infection in a Patient With Nasal Septum Perforation, Cocaine Use Disorder, and a Perinuclear Anti-neutrophil Cytoplasmic Antibody (p-ANCA)-Positive Assay

**DOI:** 10.7759/cureus.54022

**Published:** 2024-02-11

**Authors:** Rebecca O'Connell, Richard Rogers, Ian Brandon, Lorena Del Pilar Bonilla

**Affiliations:** 1 Internal Medicine, Florida International University, Herbert Wertheim College of Medicine, Miami, USA; 2 Family Medicine, Florida International University, Herbert Wertheim College of Medicine, Miami, USA; 3 Family Medicine, Baptist Health South Florida, Miami, USA; 4 Translational Medicine, Florida International University, Herbert Wertheim College of Medicine, Miami, USA; 5 Internal Medicine, Baptist Health South Florida, Miami, USA

**Keywords:** pseudomonas, cocaine, vasculitis, anca, cellulitis, substance use disorder, nasal septal perforation, substance abuse

## Abstract

Nasal septum perforation (NSP) occurs secondary to many underlying etiologies, including facial trauma, drug use, malignancy, infection, or autoimmune disease. We present the case of a 39-year-old female with a past medical history of cocaine use disorder who presented with symptoms concerning facial cellulitis unresponsive to antibiotic therapy. Physical exam and subsequent imaging revealed the presence of NSP. The patient underwent a full workup exploring potential etiologies of NSP in the setting of cocaine use disorder, with lab results indicating *Pseudomonas aeruginosa *and *Pseudomonas putida *cellulitis as well as a positive perinuclear anti-neutrophil cytoplasmic antibody (p-ANCA) assay. This case highlights the importance of maintaining a broad differential diagnosis for the etiology of NSP and avoiding anchoring bias.

## Introduction

Nasal septum perforation (NSP) is a condition in which a full-thickness defect forms between the right and left nasal cavities, typically secondary to a primary pathologic mechanism [1]. With a prevalence estimated as high as 2% in urban populations, NSP remains a significant burden to the healthcare system [2]. It is important to conduct full evaluations of these patients to address potential underlying mechanisms, since many of the said mechanisms are potentially fatal if left untreated [1].

While it is important to identify the underlying mechanism for NSP, a wide differential makes it challenging for clinicians to isolate and appropriately treat the mechanism of the perforation. The two most common etiologies include traumatic/iatrogenic sources (e.g., prolonged nasogastric tube use, status-post septoplasty, nasal bone fracture) and intranasal drug use (e.g., cocaine, intranasal steroids, chronic nasal decongestant). Less common but equally or more dangerous etiologies include inflammatory conditions (e.g., granulomatosis with polyangiitis, sarcoidosis), infections (e.g., syphilis, fungal infections), and neoplasms (e.g., nasopharyngeal carcinomas, mid-facial lymphomas) [3]. Moreover, many cases of NSP are multifactorial in nature, such as the current case in question. This furthers the diagnostic challenge of NSP.

The anchoring bias remains prevalent within the medical field, which is the tendency to latch or "anchor" onto information first presented about a patient and to rely on that information in subsequent judgements regardless of its accuracy. With this potential bias, it is ever important to fully consider all possible diagnoses of patients with NSP [4]. We present a case of NSP with concurrent *Pseudomonas aeruginosa* and *Pseudomonas putida* facial cellulitis, cocaine use disorder, and a subsequently positive perinuclear anti-neutrophil cytoplasmic antibody (p-ANCA) indirect fluorescent antibody assay. This case illustrates the importance of maintaining a broad differential and avoiding anchoring biases in cases of NSP with seemingly obvious etiologies such as cocaine-induced pseudovasculitis.

## Case presentation

This is a case of a 39-year-old female with a past medical history of epilepsy and polysubstance use disorder including cocaine. She reported using cocaine over the past two years and twice in the past month since this current admission. The patient was recently admitted in August 2023 to the hospital for facial cellulitis and treated with intravenous antibiotics including vancomycin and daptomycin. She was discharged home the following day on Bactrim (Roche, Basel, Switzerland). At the time, the nasal swab was methicillin-resistant *Staphylococcus aureus* (MRSA) positive. She reported that she completed the treatment without any significant improvement in the symptoms. 

She returned to the hospital in September 2023 complaining of left facial pain, redness, and swelling that started about one month prior. A review of the systems was negative except for headaches and subjective fevers. She also endorsed a dull retrosternal chest pain at rest that occurred sporadically over the last month. The patient was evaluated on this admission for concerns of left facial pain present for one month. Physical exam findings on admission were significant for a perforated nasal septum, left nasal mucosal swelling and congestion, left nare soft tissue bulge, erythema on the nasal bridge, edema, and tenderness below the left eye, nose, and cheek. Vital signs were stable.

Laboratory evaluation on admission revealed a white cell count of 12.400/uL, hemoglobin of 9.5 g/dL, hematocrit of 29.6%, platelet count of 596,000/uL, blood urea nitrogen of 29 mg/dL, and creatinine of 1.22 mg/dL. There were no red blood cells in the urine. Pregnancy test was negative. Her erythrocyte sedimentation rate was 106 mm/hr and C-reactive protein was 18.7 mg/dl. The human immunodeficiency virus (HIV) titers were nonreactive. On this admission, the MRSA swab was negative. Her urine toxicology screen was positive for cocaine and opiates. An iron panel showed a low vitamin B12 level, normal iron saturation, low transferrin, normal total iron, and low total iron-binding capacity. The p-ANCA performed by indirect fluorescent antibody assay was an abnormal positive speckled pattern at a dilution of 1:40. 

**Figure 1:**
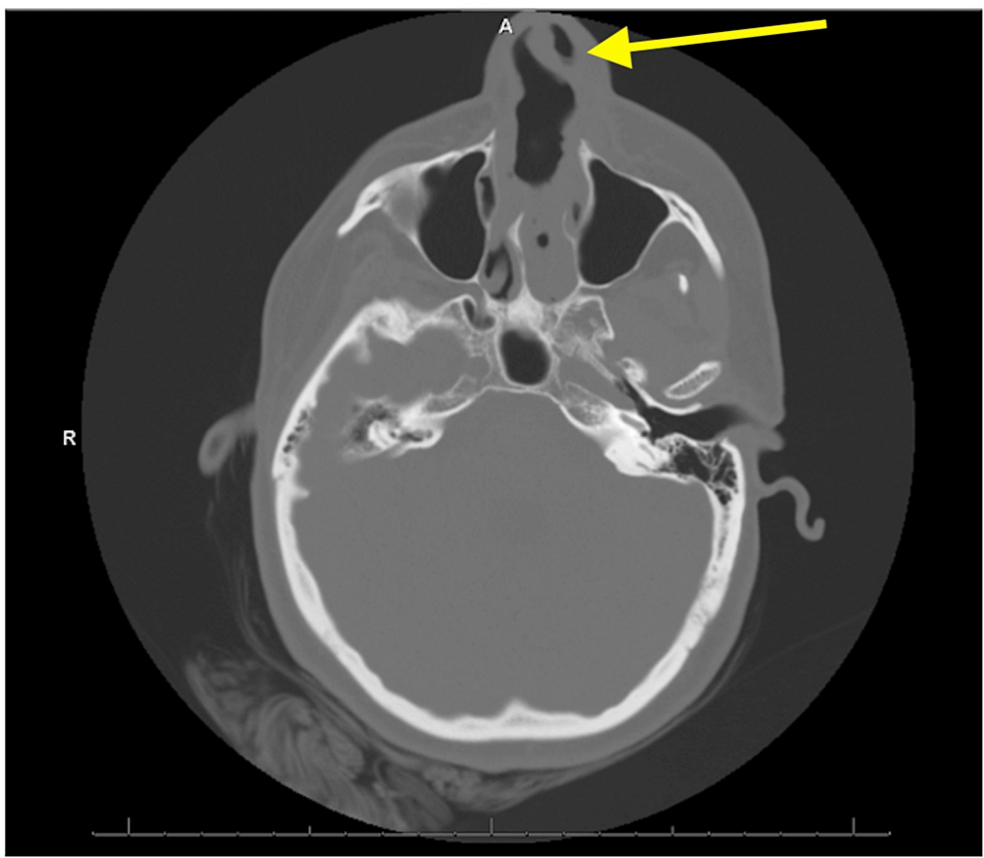
Brain CT without contrast The radiologist noted no acute intracranial abnormality but a perforated nasal septum with soft tissue thickening of the nasal region; please refer to the dedicated CT of the facial bones.

**Figure 2:**
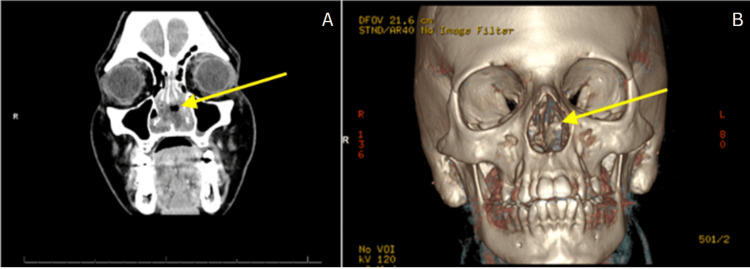
Sinus maxillofacial CT with contrast Figure 2A, per the radiology report, shows a nonspecific enhancing soft tissue within the nasal cavity associated with complete perforation of the nasal septum with overlying nasal soft tissue swelling and edema with no underlying abscess and no evidence of osteomyelitis of the underlying bone. Figure 2B is a 3D CT reconstruction demonstrating the same lesion.

A brain CT noted no acute intracranial abnormality but was significant for a perforated nasal septum with soft tissue thickening of the nasal region (Figure 1). A sinus facial maxillary CT with contrast showed complete perforation of the nasal septum with overlying nasal soft tissue swelling and edema with no underlying abscess (Figure 2). The patient was admitted for facial cellulitis in the setting of an underlying NSP in a patient with chronic cocaine use. Deep wound cultures of the nasal lesion were positive for *P. aeruginosa* and *P. putida*. Two sets of blood cultures remained sterile.

Given her reports of retrosternal chest pain, the patient underwent a cardiac evaluation. An electrocardiogram (ECG) noted normal sinus rhythm and no ST changes. A chest X-ray showed a normal cardiac silhouette and mediastinal contours. There was no pleural effusion or pneumothorax. Her lung ventilation and perfusion scan reported a low probability of pulmonary embolism. An echocardiogram noted normal left and right ventricular size, shape, and wall thickness with an estimated ejection fraction of 60-65%. There were no regional wall motion abnormalities; diastolic function, left atrial volume index, and right ventricular systolic function were normal. There was no significant pericardial effusion. An adequate jet of tricuspid regurgitation was not detected, so right ventricular systolic pressure could not be estimated. The inferior vena cava (IVC) was normal in size, and inspiratory collapse suggested a normal right atrial pressure.

Infectious disease was consulted, and the patient was started on cefepime and vancomycin. While she was hospitalized, she completed four days of cefepime and five days of vancomycin. An otolaryngologist was consulted and agreed to continue with antibiotic therapy, noting no need for debridement. A broad differential diagnosis was considered including cocaine-induced pseudovasculitis, multisystemic autoimmune disease including granulomatosis with polyangiitis, eosinophilic granulomatosis with polyangiitis, microscopic polyangiitis, relapsing polychondritis, systemic lupus erythematosus, trauma, infection, and neoplasm. 

Near the end of her stay, the patient endorsed significant improvement in erythema and tenderness. The patient was determined to be hemodynamically and clinically stable for discharge. To treat her facial cellulitis, the patient was discharged home on levofloxacin 750 mg daily for five more days per infectious disease specialist recommendations. The patient was also found to have vitamin B12 deficiency and was started on B12 replacement. Fecal occult was positive but she remained asymptomatic. Her hemoglobin was stable. She was advised to follow up with a gastroenterologist outpatient for anemia and a positive occult blood test. The patient was advised to stop cocaine use and was receptive to counseling. After discharge, the patient received a follow-up call, and she reported no pain or swelling on her face. Given her positive ANCA test, she was instructed to follow up with a rheumatologist to be evaluated for a multisystemic autoimmune condition and potential treatment with disease-modifying therapy.

## Discussion

Determining the etiology of NSP can present a challenge for clinicians, especially with the overlap in clinical presentations of cocaine-induced pseudovasculitis, inflammatory conditions (e.g., granulomatosis with polyangiitis, microscopic polyangiitis), infectious etiologies, and neoplasms. This case, in particular, highlights this challenge, as multiple potential etiologies for NSP were present, including cocaine use, facial cellulitis, and a positive p-ANCA, that may indicate an underlying inflammatory process. Out of these potential etiologies, and in light of the prevalence of cocaine use in the general population compared to rarer conditions such as granulomatosis with polyangiitis, anchoring to cocaine-induced pseudovasculitis could preclude further exploration of other possibilities [3]. 

Due to the patient's concurrent development of *P. aeruginosa* and *P. putida* cellulitis, the question of whether the cellulitis was the initiating event for the NSP development was raised. *P. aeruginosa* infections commonly present as skin manifestations such as hot tub folliculitis, toe web infection, and otitis externa [5]. However, *P. aeruginosa* does not commonly result in cellulitis, as was the case for our patient. *P. putida* is also not a common cause of cellulitis, but a 2013 case report described the first documented fatal case of *P. putida* bacteremia secondary to a soft tissue infection in an 80-year-old female [6]. Although our patient did improve with antibiotics, there is a need for further research on the diagnosis and treatment of *Pseudomonas* soft tissue infections. 

Another layer of complexity in this case was the positive p-ANCA assay, which raised concern for underlying inflammatory etiologies. ANCA-positive vasculitides like granulomatosis with polyangiitis are systemic diseases that affect multiple organs within the body. Granulomatosis with polyangiitis often presents initially with ear-nose-throat involvement (e.g., rhinitis, sinusitis, mastoiditis, saddle nose deformity, epistaxis) and can also include lung involvement (e.g., pulmonary nodules, pleural effusion) causing cough, dyspnea, wheezing, and hemoptysis [7]. A study by Cannady et al. reported NSP in 33% of their patients with granulomatosis with polyangiitis, commonly presenting as part of the initial symptomology [8]. Renal involvement is not a common primary presentation; however, 77-85% of patients ultimately develop kidney disease with signs including proteinuria and red blood cell casts. This can rapidly evolve to end-stage renal disease [7]. 

ANCA-positive vasculitides are relatively rare; however, they can be rapidly fatal if left untreated. Before corticosteroids and cytotoxic agents were used for granulomatosis with polyangiitis, the one-year mortality rate was between 50% and 80%. Once combination therapy with cyclophosphamide, azathioprine, and steroids became popular in the 1970s, ANCA-associated vasculitis achieved an 80-90% survival rate [9]. Thus, early diagnosis and treatment of ANCA-associated vasculitides is critical for lengthening survival for patients. 

Interestingly, ANCA-associated vasculitis has been attributed to levamisole, which contaminates approximately 70% of cocaine [10]. Levamisole is an antihelminthic medication that is commonly added to cocaine due to its ability to potentiate its stimulant effects. More recently, levamisole was found to have immunomodulatory properties that can present as a drug-induced ANCA-associated vasculitis with the cornerstone of treatment involving complete cessation of cocaine. The patient discussed in this report may have levamisole-induced vasculitis, which emphasizes the importance of recommending drug rehabilitation to address her cocaine addiction and further workup with a rheumatologist. 

Furthermore, there is research suggesting a disproportionate number of patients with cocaine-induced midline destructive lesions (CIMDLs) such as NSP are ANCA-positive compared to the general population. This study infers that patients who are predisposed to produce ANCAs are also more likely to develop NSP when exposed to cocaine. In the cases described in this article, the CIMDLs are clinically indistinguishable from granulomatosis with polyangiitis confined to the upper respiratory tract. Additionally, cases of CIMDL are resistant to immunosuppressive therapy, which has typically been the mainstay of treating autoimmune conditions such as granulomatosis with polyangiitis. In these cases, removal of the offending stimuli (e.g., cocaine) has been shown to limit the progression of NSP and result in more favorable post-surgical outcomes for the patient [11]. Since the patient in our case fits this category of having both a history of cocaine use and being ANCA-positive, careful consideration of her long-term treatment strategy is needed. If treated with immunosuppressant therapy, as is the standard for conditions such as granulomatosis with polyangiitis, this patient may experience inadequate response to treatment. Ultimately, cessation of cocaine use will need to be achieved to ensure lasting recovery.

## Conclusions

This paper highlights the importance of including a broad differential diagnosis for NSP. Anchoring a diagnosis such as cocaine-induced perforation can lead to the delayed diagnosis of infectious etiologies or multisystemic autoimmune conditions such as vasculitis. Early diagnosis allows for the initiation of proper treatment to prevent recurrent hospital stays and improve patient outcomes. 
